# Dissimilar Brazing of Ti–15Mo–5Zr–3Al and Commercially Pure Titanium Using Ti–Cu–Ni Foil

**DOI:** 10.3390/ma14205949

**Published:** 2021-10-10

**Authors:** Gui-Lin Yue, Tai-Cheng Chen, Ren-Kae Shiue, Leu-Wen Tsay

**Affiliations:** 1 Doctoral Degree Program in Ocean Engineering Technology, National Taiwan Ocean University, Keelung 202301, Taiwan; yueglin@163.com; 2 Engineering Training Center, Jiangsu Ocean University, Lianyungang 222005, China; 3 Division of Nuclear Fuels and Materials, Institute of Nuclear Energy Research, Taoyuan 32546, Taiwan; tcchen@iner.gov.tw; 4 Department of Materials Science and Engineering, National Taiwan University, Taipei 10617, Taiwan; rkshiue@ntu.edu.tw; 5 Department of Optoelectronics and Materials Technology, National Taiwan Ocean University, Keelung 202301, Taiwan

**Keywords:** dissimilar brazing, titanium alloy, eutectoid, dissolution, microstructure

## Abstract

Dissimilar brazing of Ti–15Mo–5Zr–3Al (Ti-1553) to commercially pure titanium (CP-Ti) using Ti–15Cu–15Ni foil was performed in this work. The microstructures in different sites of the brazed joint showed distinct morphologies, which resulted from the distributions of Mo, Cu, and Ni. In the brazed zone adhered to the Ti-1553 substrate, the partitioning of Mo from the Ti-1553 into the molten braze caused the formation of stabilized β-Ti without Ti_2_Cu/Ti_2_Ni precipitates. In the CP-Ti side, the brazed joint displayed a predominantly lamellar structure, composed of the elongated primary α-Ti and β-transformed eutectoid. The decrease in the Mo concentration in the brazed zone caused the eutectoid transformation of β-Ti to Ti_2_Cu + α-Ti in that zone. The diffusion of Cu and Ni from the molten braze into the CP-Ti accounted for the precipitation of Ti_2_Cu/Ti_2_Ni in the transformed zone therein. The variation in the shear strength of the joints was related to the amount and distribution of brittle Ti_2_Ni compounds. Prolonging the brazing time, the wider transformed zone, consisting of coarse elongated CP-Ti interspersed with sparse Ti_2_Ni precipitates, was responsible for the improved shear strength of the joint.

## 1. Introduction

Titanium alloys are featured with a high specfic strength–weight ratio and resistance to corrosion, so they are extensively used in the aerospace, marine, and automobile industries. The manufacture of Ti alloys has drawn more attention in recent years [1,2,3]. β-Ti alloys are known to have better cold workability than α-Ti alloys [4]. The combination of excellent strength, good ductility, and weldability makes β-Ti alloys very useful as structural materials in various industries [5]. Ti–15Mo–5Zr–3Al (Ti-1553), a promising β-Ti, was developed by Kobe steel for a wide range of applications [6]. The microstructures and mechanical properties of Ti-1553 change obviously with variations in heat treatment or thermo-mechanical processing [6]. After being solutionized and aged at different temperatures, the Ti-1553 alloy can achieve a combination of high strength and moderate ductility [7,8]. Moreover, it is considered to be a promising β-Ti alloy for biomedical implants because of its superior mechanical strength [9]. 

Titanium alloys can be joined by various industrial practices including diffusion bonding, welding, and brazing processes [10,11,12,13]. However, the distortion induced by welding and the geometry limitations of the components may restrict the use of fusion-welding processes [11]. Brazing is the alternative for joining different components of titanium and its alloys [14,15]. For the brazing of titanium alloys, Ti-, Ag- and Al-based fillers have been developed for industrial applications [16,17,18,19]. It is reported that the brazed joints of titanium alloys have poor bonding strength and corrosion resistance if Ag- or Al-based fillers are applied [17,18]. Due to its good brazing ability and high bonding strength, the Ti–Cu–Ni alloy is used widely as a filler metal for the brazing of titanium alloys [20]. As reported previously, complex intermetallic compounds can form in a titanium alloy joint alloyed with Ni and Cu [10,20]. 

The open literature reports many studies focusing on the brazing of similar titanium alloys [21,22], but less attention has been paid to the brazing of dissimilar ones [23]. Brazing the β-Ti alloy (Ti–15Mo–5Zr–3Al) with clad Ti–15Cu–15Ni filler foil has been investigated [20]. The mass transport of Cu and Ni in β-Ti plays an important role in forming Ti–Cu–Ni intermetallic compounds in the brazed zone, and it is strongly related to the bonding strength of the joint. The tendency of Ti–Cu–Ni intermetallics to form in β-Ti is quite different from that in α-Ti due to the much higher solubilities of Cu and Ni in the β-Ti substrate. The mechanism of the formation of distinct intermetallic compounds in a joint composed of α- and β-Ti substrates and brazed with Ti–Cu–Ni filler needs further investigation [24]. To date, the interfacial reactions between the filler and β-Ti in the joint, and those between the filler and α-Ti have received little study. Commercially pure Ti (CP-Ti, α-Ti) is used for different applications [25]. Dissimilar brazing of CP-Ti and β-Ti is an appropriate combination for the exploration of the mechanism of the formation of intermetallic compounds in two types of titanium alloys. Therefore, this study focused on the interfacial reactions between CP-Ti (α-Ti) and Ti-15553 (β-Ti) in a joint brazed with Ti–Cu–Ni foil. The microstructural evolution of the dissimilar joint was investigated in terms of the diffusion of copper and nickel, and the results were related to the change in shear strength of the joint. A scanning electron microscope (SEM) and an electron probe microanalyzer (EPMA) were employed to examine the microstructures and measure the chemical compositions. Electron backscattered diffraction (EBSD) was applied to distinguish various phases in the joint.

## 2. Materials and Methods

The dimensions of the substrate used in this work were 10 × 10 × 3 mm^3^. The chemical compositions in at% of the tested materials are listed in Table 1. All brazed samples were ground with 100, 320, and 600 grit sand papers prior to the brazing experiments. A clad Ti–Cu–Ni foil of 60 μm in thickness was used as the filler in this work. The substrate and filler were cleaned ultrasonically in ethanol for 15 min prior to being placed in the vacuum furnace. The brazed sample was constructed in a sandwiched configuration with the filler sandwiched between the Ti-1553 and CP-Ti. A graphite fixture was used to hold the assembled sample to prevent distortion during brazing. The fixture was subsequently installed in the furnace at a vacuum of 5 × 10^−5^ Pa then preheated to 1073 K at a heating rate of 20 K/min. The samples were brazed at 1243 K for 3, 30, and 60 min, respectively.

**Figure 1:**
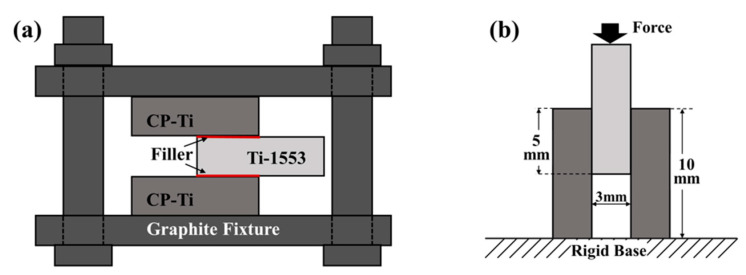
Schematic diagrams of (**a**) double lap joints, (**b**) the dimensions of the sample for the shear test.

The brazed joints received metallographic treatments after the brazing cycle was completed. The brazed joints were sectioned with a slow speed cutter then ground and polished for microstructural examinations. A Hitachi 3400 SEM (Hitachi Ltd., Tokyo, Japan) in backscattered electron (BSE) image was used to examine the details of the microstructures in distinct zones of the brazed joints. Quantitative chemical analysis of selected phases in the brazed joint at specific locations were examined with an electron probe micro-analyzer (EPMA, JEOL JXA-8200, JEOL Ltd., Tokyo, Japan). An SEM (JSM-7100F, JEOL, Tokyo, Japan) equipped with the electron backscattered diffraction detector (EBSD, Oxford Instruments, Abingdon, UK) was applied to investigate distinct phases in the joint. Double lap joints, CP-Ti/Filler/Ti-1553/Filler/CP-Ti were constructed to measure the shear strength (Figure 1a). A tensile instrument (AG-10, Shimadzu Crop., Kyoto, Japan) was applied to measure the shear strength at a compression rate of 1 mm/min (Figure 1b). The shear strength of the brazed joint was counted by the applied peak load divided by the brazed area. At least three samples for each test were used to calculate the average joint strength and the standard deviation in data was included. The shear fractured samples were cut into small pieces and subjected to metallographic preparations before examining the fracture path. The fracture features and paths were inspected by SEM, and the fracture mechanism of the brazed joint was proposed.

## 3. Results and Discussion

### 3.1. Microstructural Analyses

**Figure 2:**
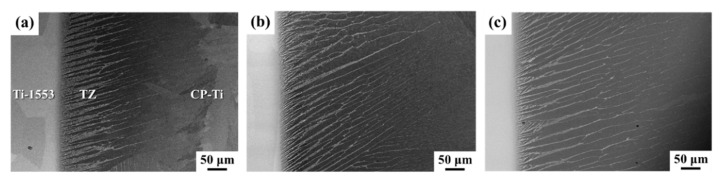
SEM-BEI (back-scattered electron image) photos of Ti-1553/Ti–15Cu–15Ni/CP-Ti brazed at 1243 K for (**a**) 3 min, (**b**) 30 min, and (**c**) 60 min.

Figure 2 presents the SEM-BEI photos of the Ti-1553/Ti–Cu–Ni/CP-Ti joints brazed at 1243 K for 3, 30, and 60 min, respectively. The microstructures of the dissimilar brazed joint showed different morphologies at distinct sites. The microstructure of the Ti-1553 side was quite different from that of the CP-Ti side. No second phase was observed in the Ti-1553 side. However, a lamellar structure with a width of about 250 μm initiated from the brazed zone and propagated toward the CP-Ti substrate in the tranformed zone (TZ), as indicated in Figure 2a. Increasing the brazing time, the lamellar structure increased in length, as can be seen by comparing Figure 2a–c. The growth of the lamellar plate was related to the diffusion of Cu and Ni into the CP-Ti substrate and will be discussed later in the text.

**Figure 3:**
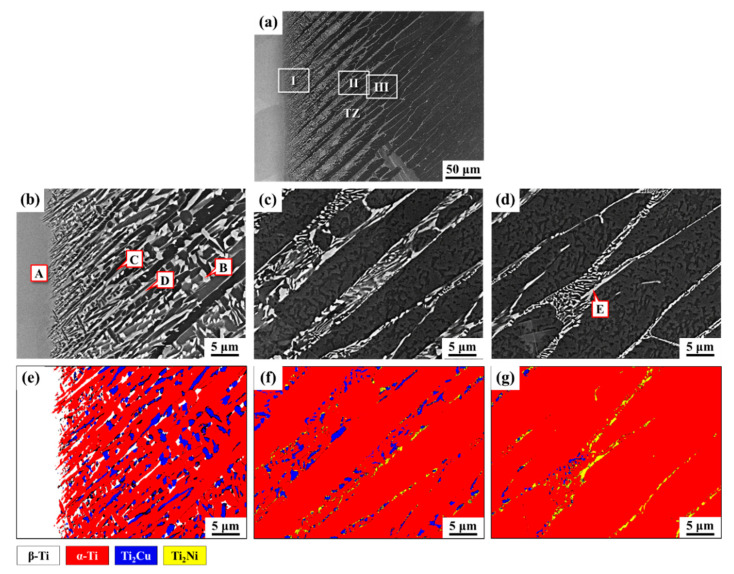
SEM-BEI photos and EBSD maps of Ti-1553/Ti–Cu–Ni/CP-Ti joint brazed at 1243 K for 3 min: (**a**) cross section overview; (**b**–**d**) higher magnification of location I, II, III in 3a; (**e**–**g**) EBSD phase maps of 3b to 3d.

Figure 3 shows the SEM-BEI photos and EBSD phase maps of a Ti-1553/Ti–Cu–Ni/CP-Ti joint brazed at 1243 K for 3 min. Selected areas I, II, and III are provided at higher magnification in Figure 3b–d. Quantitative chemical EPMA analysis at positions A to E (Figure 3b–d) are listed in Table 2. Figure 3e–g are the EBSD phase maps of Figure 3b–d, respectively. According to the EBSD maps, four phases, β-Ti (white), α-Ti (red), Ti_2_Cu (blue), and Ti_2_Ni (yellow), coexisted in the dissimilar brazed joint. In region I, as listed in Table 2 and Figure 3e, the brazed zone next to the Ti-1553 side was a typical β-Ti marked A in Figure 3a. The brazed zone of the CP-Ti side consisted of eutectoid Ti_2_Cu (marked B) + α-Ti (marked C), and retained β-Ti (marked D). In Table 2, the chemical composition of Ti_2_Cu shows that it was alloyed with a high Ni concentration. This is consistent with the Cu–Ni–Ti ternary alloy phase diagram at 1073 K; the intermetallic Ti_2_Cu was alloyed with Ni of up to 13 at% [26]. In addition, the molten braze solidified into β-Ti, which was alloyed with Cu, Ni, Al, Mo, and Zr. The occurrence of the subsequent eutectoid decomposition caused β-Ti to transform into α-Ti + Ti_2_Cu during the cooling cycle of the brazing. In addition, the segregation of β stabilizing elements accounted for the formation of a little β-Ti retained in the lamellar structure, as revealed in Figure 3b,e.

The thickness of the Ti–Cu–Ni filler foil was 60 μm. In region I (the brazed zone) in Figure 3a, dense and fine precipitates were separated by a dark elongated phase. In Figure 3a, the growth of the lamellar structure into the CP-Ti substrate can be clearly observed. From regions II and III in Figure 3a, it appears that the microstructure of TZ comprised of elongated primary α-Ti and transformed β-Ti oriented in lamellar form (Figure 3c,d). The reaction of the eutectoid assisted the decomposition of β-Ti into predominantly α-Ti + Ti_2_Cu and a few Ti_2_Ni precipitates (Figure 3f). It is noted that a little retained β-Ti was observed in region II (Figure 3f), which could be attributed to the limited Mo content. With increases in the penetration depth into the CP-Ti substrate, the microstructure of region III (Figure 3d) showed coarsening of the primary α-Ti compared with those in region II. Furthermore, the volume fraction of eutectoid α-Ti + Ti_2_Cu in the joint was significantly lower in region III than that in region II. It is noted that thin Ti_2_Ni films, marked E in Figure 3d, were located between the elongated α-Ti. Regarding the intermetallics, the amount of Ti_2_Ni was greater than that of Ti_2_Cu farther from the brazed zone and closer to the CP-Ti side, as illustrated in Figure 3g. 

**Figure 4:**
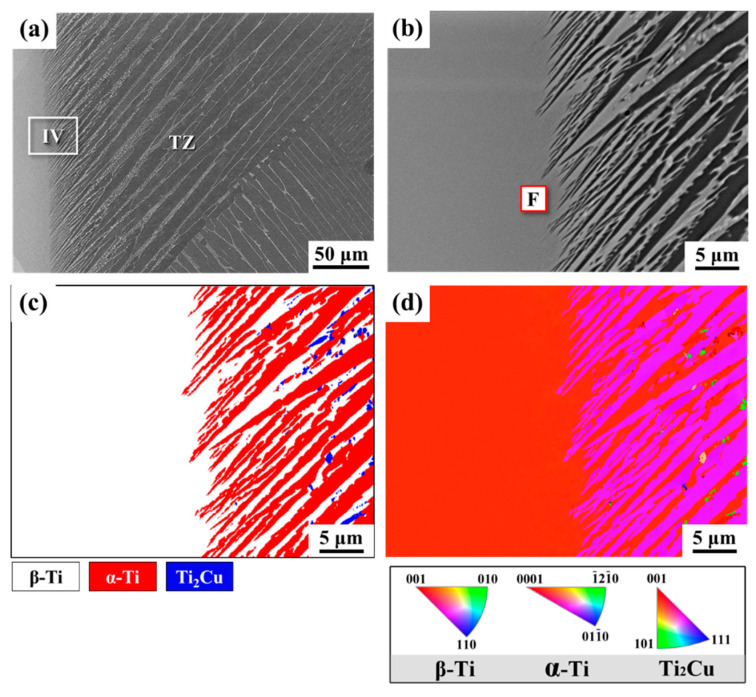
SEM-BEI photos and EBSD maps of Ti-1553/Ti–Cu–Ni/CP-Ti joint brazed at 1243 K for 30 min: (**a**) cross-section overview; (**b**) higher magnification of the region IV in 4a; (**c**,**d**) EBSD phase map and inverse pole figure (IPF) map of 4b.

Figure 4 shows the SEM-BEI photos and EBSD maps of Ti-1553/Ti–Cu–Ni/CP-Ti joints brazed at 1243 K for 30 min. A selected area in the brazing zone, marked IV, is displayed at higher magnification in Figure 4b. The chemical composition at position F (Figure 4b) is listed in Table 2. Figure 4c,d show the EBSD phase map and inverse pole figure (IPF) in Figure 4b, respectively. Based on Figure 4b, the microstructure in the brazing zone mainly consisted of fine lamellar α-Ti, β-Ti, and a few Ti_2_Cu. The different phases in the brazed zone were verified by the phase map, as illustrated in Figure 4c. The β-Ti was stabilized by alloying with 4.9 at% Mo due to the dissolution of the Ti-1553 substrate, marked F in Figure 4b and Table 2. It was noted that the amount of Ti_2_Cu decreased near the interface with increasing brazing time, as can be seen from comparing Figure 4c,e. As shown in Figure 4d, the β-Ti (red) and the α-Ti (purple) were oriented in a specific direction. If the temperature is lowered past the β-transus temperature, the growth of primary α-Ti from the parent β-Ti can be expected to follow the fixed relationship [27]. 

**Figure 5:**
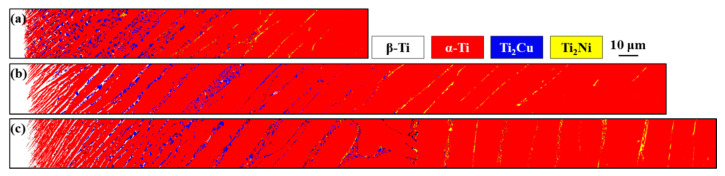
EBSD crystallographic analysis results of Ti-1553/Ti–Cu–Ni/CP-Ti joint brazed at 1243 K for (**a**) 3, (**b**) 30, and (**c**) 60 min.

Figure 5 shows the EBSD phase maps of the joint brazed at 1243 K for different durations to investigate the phase evolution of the joint. The results revealed that the width of the TZ increased with increasing brazing time. Moreover, the density of the Ti_2_Cu precipitates close to the Ti-1553 side became sparse in distribution with increases in brazing time, which resulted fom the redistribution of Mo, Cu, and Ni across the joint. The variation in the joint microstructure was related mainly to the high-temperature partitioning and diffusion of alloying elements in the brazed zone. 

Mo is known to be a strong isomorphous β stabilizer [28]. When the Mo concentration is sufficiently high, the stable phase at ambient temperature will be β-Ti, so β-Ti will form in the joint. Cu and Ni are the eutectoid β forming elements. The maximum solubilities of Cu and Ni in β-Ti were 13.5 at% and 10 at%, respectively [28]. In the brazed zone adhered to Ti-1553, the thin β-Ti was stabilized by the partitioning of Mo from the Ti-1553 substrate and into the brazed zone. The high solubility of the β stabilizers (i.e., Mo, Cu and Ni) in the β-Ti was responsible for the lack of Ti_2_Cu/Ti_2_Ni precipitates in the brazed zone next to the Ti-1553. 

Further into the CP-Ti from the stabilized β film, the elongated α-Ti was the major phase dominating the brazed zone. In the specimen brazed for 3 min, most of the Cu and Ni was retained in the brazed zone. The lack of high concentration Mo partitioning led to the formation of a few retained β-Ti in the brazed zone close to the CP-Ti side. Both primary α-Ti and eutectoid Ti_2_Cu + α-Ti dominated the brazed zone, as illustrated in Figure 5a. Prolonging the brazing time enhanced the formation of film-like retained β-Ti (white) in the brazed zone of the CP-Ti side, as displayed in Figure 5b,c. With the enhanced diffusion of Cu and Ni from the brazed melt into the CP-Ti side, the eutectoid Ti_2_Cu + α-Ti and Ti_2_Ni in between the primary α-Ti were obtained in the transformed zone. Far from the brazed zone, the smaller Cu/Ni concentration accounted for the formation of coarser primary α-Ti and few precipitates. The β-Ti alloyed with Cu decomposed into eutectoid Ti_2_Cu and α-Ti. It is worth noting that in β-Ti, Ni atoms diffuse faster than Cu. Notably, the eutectoid was replaced by Ti_2_Ni in between the boundaries of primary α-Ti close to the CP-Ti side. This change was much more obvious in the specimen brazed for longer periods (e.g., 60 min).

### 3.2. Shear Failure Analyses

Table 3 lists the average shear strengths of the joints brazed at 1243 K for various time intervals. When the holding time was 3 min, the average shear strength of 275 MPa with the minimum standard deviation of 4 MPa was the lowest among the tested samples. The average shear strength of the joint increased with prolongation of the brazing time. The maximum shear strength of 315 MPa was achieved in the specimen brazed for 60 min.

**Figure 6:**
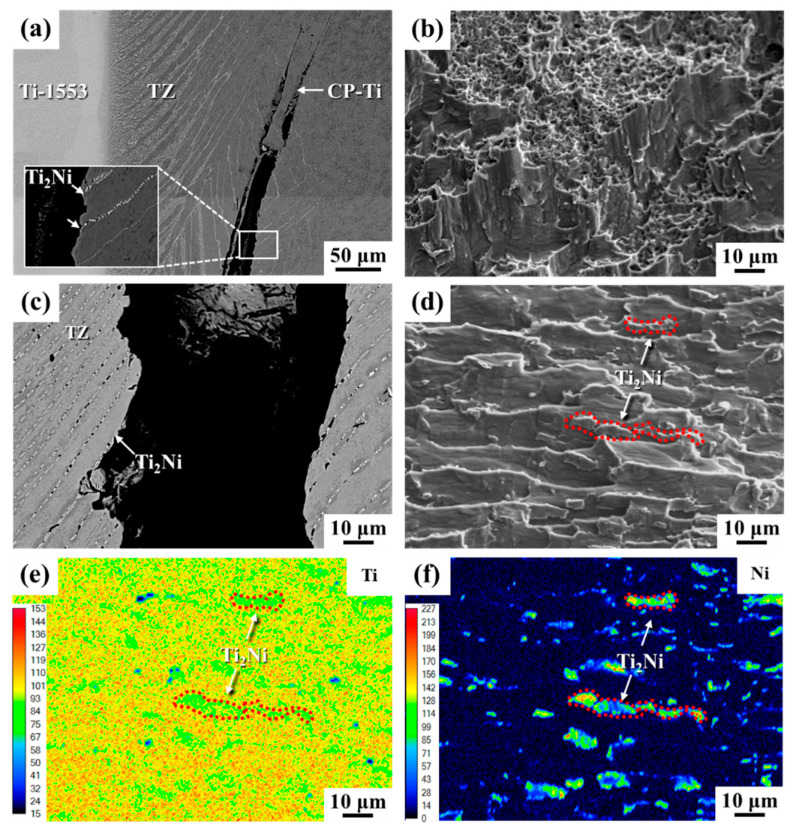
Cross-sectional view of the fractured joint in the SEM-BEI images and shear fracture feature of the brazed joint brazed for (**a**,**b**) 3 min, (**c**,**d**) 60 min; (**e**,**f**) the Ti and Ni element mappings of the fracture surface in 6d.

Figure 6 shows the failure characteristics of the brazed joints after shear tests. It was expected that the Ti_2_Ni intermetallic compound would be very brittle and that cracks would be easily initiated if the external loading was applied. According to the cross-sectional views shown in Figure 6a,c, the cracks were prone to initiate at and propagate along the Ti_2_Ni intermetallics in the transformed zone. In the sample brazed for 3 min, the main crack was found to nucleate in the transformed zone close to the CP-Ti substrate (Figure 6a). The deflection of the crack growth path caused the final fracture to be located at the weaker CP-Ti side. It was deduced that the narrow transformed zone can not easily restrict the crack growth direction. The fracture features were mainly ductile dimple mixed with tear shear (Figure 6b), but the shear strength was relatively low. Prolonging the brazing time caused the Ni atoms to diffuse deeper into the CP-Ti substrate, resulting in forming a wider transformed zone with aligned Ti_2_Ni precipitates. As shown in Figure 6c, the microcracks tended to nucleate at the Ti_2_Ni precipitates and propagate along the aligned Ti_2_Ni. The fracture displayed aligned tear ridges with precipitates on them (indicated by the arrows in Figure 6d). Ti and Ni mappings of the fracture surface by EPMA are shown in Figure 6e,f. It was confirmed that the cracks propagated through the aligned Ti_2_Ni precipitates in between the lamellar α-Ti. The coarse lamellar structure, comprising coarse primary α-Ti and thin layers of Ti_2_Ni, led to a reduction in the brittleness and an increase in the shear strength of the brazed joints with longer brazing times.

### 3.3. Schematic Diagram of the Brazing Process

**Figure 7:**
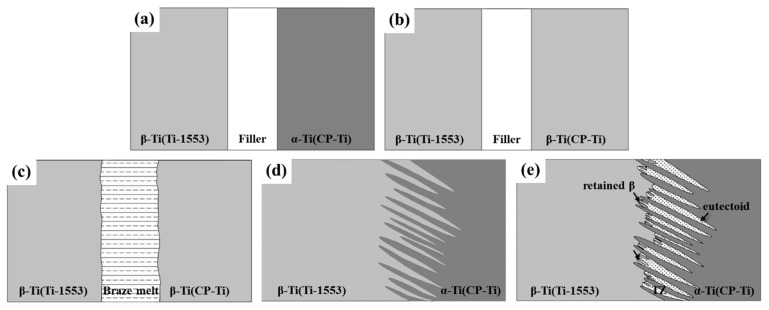
Schematic diagrams of microstructural evolution at different stages of brazing: heating stage, (**a**) RT; (**b**) T_βtrans_ < T < T_brazing_, brazing stage; (**c**) T_brazing_, cooling stage; (**d**) T_eutectoid_ < T < T_βtrans_; (**e**) T < T_eutectoid_.

Figure 7 presents the microstructural evolution at distinct stages of brazing. When the furnace was heated to a temperature lower than the beta-transus (T_β__trans_), the substrate and the filler remained in their initial microstructures (Figure 7a). While heating above the beta-transus temperature but below the melting point of the filler, the α-Ti to β-Ti transformation occurred in the CP-Ti substrate (Figure 7b). During brazing, both sides of the substrates were stable β-phase, whereas the filler melted (Figure 7c). The dissolution of the substrate into the molten braze and the diffusion of Cu/Ni to the substrate resulted in the isothermal solidification of the molten braze and the formation of solidified β-Ti in the brazed zone. During the cooling stage, in the brazed zone adhered to the Ti-1553 substrate, the Mo concentration was high enough to stabilize β-Ti at room temperature. On the CP-Ti side, when the temperature was cooled down to below the beta-transus, primary α-Ti nucleated and grew from the β phase (Figure 7d). However, a few β-Ti between the primary α-Ti were un-transformed by the alloying of Mo, Cu, and Ni. While cooling below the eutectoid transformation temperature, β-Ti would decompose into Ti_2_Cu/Ti_2_Ni + α-Ti (Figure 7e). If the concentration of β stabilizer is sufficiently high, the few retained β-Ti (marked D in Figure 3b) will be stabilized in between the primary α-Ti at ambient temperature after the invariant reaction (Figure 7e). 

## 4. Conclusions

Dissimilar brazing of Ti–15Mo–5Zr-3Al and CP-Ti was carried out with Ti–15Cu–15Ni foil. Important results are included as follows:

(1) The partitioning of Mo from the Ti-1553 substrate into the molten braze enhanced the formation of a stable β-Ti film, which adhered to the Ti-1553 substrate in the joint. The enrichment of Cu and Ni in the brazed zone caused the formation of a lamellar structure, composed of elongated primary α-Ti and β-transformed eutectoid of β-Ti. The microstructure of the β-transformed eutectoid showed predominantly lamellar α-Ti + Ti_2_Cu and a little retained β-Ti.
(2) Increasing the brazing time from 3 to 60 min caused the dense Ti_2_Cu precipitates located near the joint interface to become sparse in distribution, in conjunction with coarsening of the elongated primary α-Ti by the diffusion Cu/Ni into the CP-Ti substrate. The diffusion distance of Ni into the CP-Ti substrate was longer than that of Cu, as demonstrated by the presence of the Ti_2_Ni/α-Ti eutectoid.
(3) The results of shear tests showed that the cracks tended to initiate at and propagate along the Ti_2_Ni intermetallics in the transformed zone. Due to the narrow transformed zone, the deflection of the crack growth path caused the final fracture to be located at the weaker CP-Ti side when the brazing time of 3 min was applied. The shear fracture features showed mainly ductile dimple mixed with tear shear, but a lower shear strength was obtained for samples with shorter brazing times. In the specimen brazed for 60 min, the wider transformed zone consisted of coarse elongated CP-Ti interspersed with sparse Ti_2_Ni precipitates. The decrease in the brittleness of the brazed joint was responsible for the increase in shear strength.

## Figures and Tables

**Table 1:** Nominal compositions in at% of Ti–15Mo–5Zr–3Al and Ti–15Cu–15Ni.

Material/at%	Ti	Cu	Ni	Mo	Zr	Al
Ti–15Mo–5Zr–3Al	83.8	---	---	7.8	2.8	5.6
Ti–15Cu–15Ni	74.8	12.1	13.1	---	---	---

**Table 2:** Chemical compositions of positions A–F in Figure 3b–d and Figure 4b.

Location/(at%)	Ti	Cu	Ni	Al	Mo	Zr	Phase
A	86.6	2.6	3.5	2.2	4.1	1.0	β-Ti
B	70.5	16.0	12.4	0.4	0.3	0.4	Ti_2_Cu
C	98.3	0.4	0.2	0.9	0.0	0.2	α-Ti
D	86.1	2.2	5.5	1.6	3.6	1.0	β-Ti
E	77.4	1.6	20.9	0.1	0.0	0.0	Ti_2_Ni
F	87.8	1.6	1.9	2.7	4.9	1.1	β-Ti

**Table 3:** Shear strengths of brazed joints for three brazing temperatures.

Temperature	Holding Time	Average Shear Strength
1243 K	3 min	275 ± 4 MPa
	30 min	294 ± 10 MPa
	60 min	315 ± 8 MPa

## Data Availability

Not applicable.
